# Far‐Infrared Hyperbolic Phonon–Polaritons in Zirconium Disulfide

**DOI:** 10.1002/adma.74154

**Published:** 2026-08-06

**Authors:** Subhodip Saha, Ryan Kowalski, Joseph R. Matson, Thomas G. Folland, Tony Low, Joshua D. Caldwell, In‐Ho Lee, Sang‐Hyun Oh

**Affiliations:** ^1^ Department of Electrical and Computer Engineering University of Minnesota Minneapolis Minnesota USA; ^2^ Department of Mechanical Engineering Vanderbilt University Nashville Tennessee USA; ^3^ Interdisciplinary Materials Science Program Vanderbilt University Nashville Tennessee USA; ^4^ Department of Physics and Astronomy The University of Iowa Iowa City Iowa USA; ^5^ Center for Quantum Technology Post‐Silicon Semiconductor Institute Korea Institute of Science and Technology Seoul Republic of Korea; ^6^ Division of Advanced Materials Science and Engineering KIST School at University of Science and Technology Seoul Republic of Korea

**Keywords:** far‐infrared spectroscopy, hyperbolic phonon polaritons, light–matter interactions, transition metal dichalcogenides

## Abstract

Group‐IVB transition‐metal dichalcogenides (TMDs) have recently emerged as a promising material platform for extreme light confinement, with Hf‐based compounds demonstrating confinement factors exceeding two orders of magnitude in the far‐infrared. As a complementary Zr‐based member of this material family, zirconium disulfide (${\mathrm{ZrS}}_{2}$) combines a comparably broad first Reststrahlen band with semiconducting electronic character, providing a wide spectral window for phonon‐dominated far‐infrared hyperbolic polaritonics. Here, we report the first experimental demonstration of far‐infrared hyperbolic phonon polaritons in the group‐IVB TMD ${\mathrm{ZrS}}_{2}$ using a resonator‐assisted far‐field spectroscopy platform. An unpatterned ${\mathrm{ZrS}}_{2}$ flake integrated with a metallic ribbon array forms a phonon polariton resonator, enabling efficient far‐field excitation of phonon polaritons while suppressing extrinsic scattering losses. This high coupling efficiency enables far‐field observation of multiple polaritonic resonances beyond the fundamental branch. The large normalized light–matter coupling strength of ${\mathrm{ZrS}}_{2}$ enables ultrahigh in‐plane momenta, with effective refractive indices as high as 223. Despite this extreme confinement, linewidth analysis indicates that the measured damping is primarily governed by intrinsic propagation loss, corresponding to a sub‐picosecond polariton lifetime. These results establish ${\mathrm{ZrS}}_{2}$ as a van der Waals hyperbolic material platform for ultraconfined far‐infrared phonon polaritons and highlight the potential of group‐IVB TMDs for compact far‐infrared nanophotonic and thermal photonic applications.

## Graphical abstract

An unpatterned ${\mathrm{ZrS}}_{2}$ flake, coupled to far‐field infrared light by metal nanoribbons, reveals hyperbolic phonon polaritons with multiple higher‐order branches and effective refractive indices up to 223. This resonator‐assisted approach establishes ${\mathrm{ZrS}}_{2}$ as a compact van der Waals platform for deeply subwavelength far‐infrared confinement, thermal photonics, and nanophotonic devices.

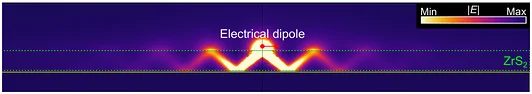

## 1 Introduction

Phonon polaritons are hybrid light–matter excitations arising from the coupling between electromagnetic fields and optical phonons in polar crystals [1, 2, 3, 4]. In anisotropic polar crystals, when the principal permittivity components have opposite signs along different crystallographic directions, these excitations can form hyperbolic phonon polaritons (HPhPs) characterized by open hyperbolic isofrequency contours [5, 6, 7, 8, 9]. Several van der Waals polar materials, including hexagonal boron nitride (hBN) [1, 5, 7, 10, 11], vanadium oxide (α‐$V_{2}O_{5}$) [12], gallium selenide (ε‐GaSe) [13], and molybdenum trioxide (${\mathrm{MoO}}_{3}$) [14, 15, 16], have been shown to support HPhPs. Recent studies have expanded the control of hyperbolic polaritons beyond simple directional propagation, demonstrating source‐configured symmetry‐broken polaritons [17], switchable focusing of hyperbolic polariton rays [18], and asymmetric polariton propagation [19]. Related work has also shown that hyperbolic surface phonon polaritons can emerge on non‐hyperbolic crystal surfaces, highlighting broader opportunities for engineering polariton topology beyond intrinsically hyperbolic media [20].

A defining characteristic of HPhPs is their ability to confine electromagnetic fields to deeply subwavelength dimensions [2, 3]. Compared with hyperbolic plasmon polaritons [21, 22], which exhibit phenomenologically similar behavior, HPhPs can achieve comparable spatial confinement while exhibiting intrinsically low optical losses [8, 23]. This low‐loss nature originates from the long lifetimes of optical phonons, typically on the order of picoseconds [24, 25].

HPhPs exist within a specific spectral window bounded by the transverse optic (TO) phonon frequency and the longitudinal optic (LO) phonon frequency, commonly referred to as the Reststrahlen band, where the real part of the dielectric permittivity becomes negative. For many polar materials, the Reststrahlen band resides in the mid‐infrared (mid‐IR) spectral range [26]. In contrast, layered group‐IVB transition metal dichalcogenides (TMDs) [27, 28, 29, 30] can support HPhPs in the far‐infrared (far‐IR) regime [31]. Recent works have demonstrated ultraconfined far‐IR HPhPs in ${\mathrm{HfS}}_{2}$ and ${\mathrm{HfSe}}_{2}$ with polariton wavelengths approximately 250 times smaller than the free‐space wavelength [32]. In these materials, unusually large LO–TO phonon splittings arise from substantial Born effective charges [33]. Combined with their intrinsically low optical phonon frequencies, the large LO–TO phonon splitting results in strong light–matter interactions that can exceed those observed in conventional mid‐IR polar materials. The deeply subwavelength confinement and enhanced light–matter interaction enabled by far‐IR HPhPs are particularly attractive for thermal photonics [34, 35], surface‐enhanced infrared spectroscopy [36, 37], and compact polaritonic components [38, 39, 40], where nanoscale control of low‐energy infrared radiation is essential.

Despite these promising attributes, group‐IVB TMDs (Ti‐, Zr‐, and Hf‐based compounds) have received significantly less attention than their group‐VIB counterparts, which have historically dominated studies of two‐dimensional semiconductors [41]. From a lattice‐dynamical perspective, however, the distinction between these two material classes is particularly pronounced. Group‐VIB TMDs, such as ${\mathrm{MoS}}_{2}$, ${\mathrm{MoSe}}_{2}$, ${\mathrm{WS}}_{2}$, and ${\mathrm{WSe}}_{2}$, exhibit relatively narrow Reststrahlen bands due to their smaller Born effective charges and correspondingly weak LO–TO phonon splitting, which makes the observation of phonon polaritons in these materials intrinsically challenging.

In contrast, zirconium disulfide (${\mathrm{ZrS}}_{2}$), together with other group‐IVB TMDs such as ${\mathrm{TiS}}_{2}$ and ${\mathrm{HfS}}_{2}$, crystallize in a trigonal, layered ${\mathrm{CdI}}_{2}$‐type structure, characterized by mixed ionic–covalent metal–chalcogen bonding within each layer and weak van der Waals interactions along the *c*‐axis [29]. This bonding character, together with the intrinsic crystallographic anisotropy, is associated with large lattice polarizability and Born effective charges, leading to strong infrared‐active optical phonons. As a result, group‐IVB TMDs exhibit pronounced LO–TO phonon splitting and substantially broader Reststrahlen bands at low photon energies, rendering them particularly favorable platforms for supporting HPhPs in the far‐IR regime. Among these materials, ${\mathrm{ZrS}}_{2}$ occupies a particularly attractive yet unexplored regime, combining semiconducting character with strong lattice anisotropy and soft infrared‐active phonons [42, 43, 44, 45]. This semiconducting character helps avoid the strong free‐carrier screening expected in ${\mathrm{TiS}}_{2}$, whose electronic character is often described as small‐gap or semimetallic [29, 46, 47].

Here, we experimentally demonstrate far‐IR HPhPs in the group‐IVB TMD ${\mathrm{ZrS}}_{2}$ using a resonator‐assisted far‐field spectroscopy approach. By integrating an unpatterned ${\mathrm{ZrS}}_{2}$ flake with a proximal metal ribbon array, we achieve efficient far‐field excitation of HPhPs while minimizing extrinsic scattering and surface‐roughness‐induced losses. The high coupling efficiency, together with the low scattering loss, enables the observation of higher‐order resonances in a far‐field measurement configuration. Through systematic comparison with full‐wave electromagnetic simulations, we identify the physical origin of these resonances and extract the corresponding polariton dispersion and damping characteristics, revealing ultrahigh in‐plane momenta as well as intrinsically low propagation loss.

## 2 Results and Discussion

### 2.1 Hyperbolic Nature of Phonon Polaritons in ${\mathrm{ZrS}}_{2}$

**FIGURE 1:**
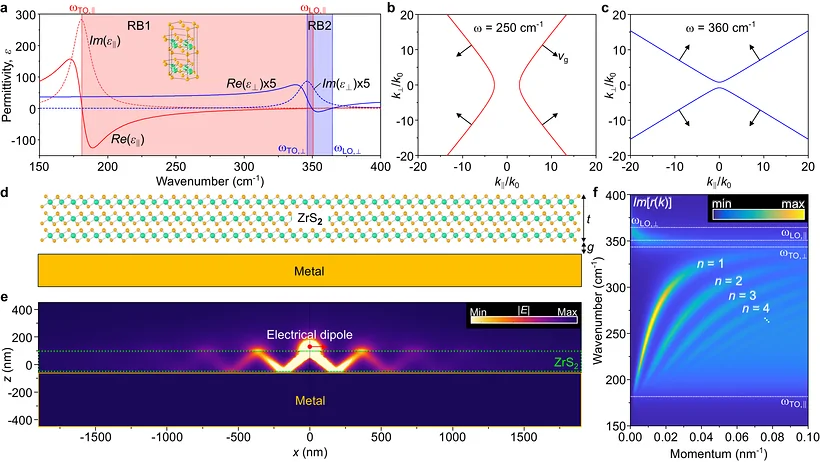
Hyperbolic nature of phonon polaritons in ${\mathrm{ZrS}}_{2}$. (a) In‐plane ($\varepsilon_{∥}$) and out‐of‐plane ($\varepsilon_{⊥}$) principal permittivity components of ${\mathrm{ZrS}}_{2}$. The inset shows the crystal structure of ${\mathrm{ZrS}}_{2}$. The out‐of‐plane permittivity curves are magnified by a factor of five for visibility. $\omega_{\mathrm{TO},∥}$ and $\omega_{\mathrm{LO},∥}$ denote the in‐plane transverse and longitudinal optical phonon frequencies, respectively, while $\omega_{\mathrm{TO},⊥}$ and $\omega_{\mathrm{LO},⊥}$ denote the corresponding out‐of‐plane transverse and longitudinal optical phonon frequencies. Iso‐frequency contours at representative wavenumbers of (b) 250 ${\mathrm{cm}}^{-1}$ in the first Reststrahlen band (RB1) and (c) 360 ${\mathrm{cm}}^{-1}$ in the second Reststrahlen band (RB2). The arrows indicate the group‐velocity direction. (d) Schematic of a van der Waals ${\mathrm{ZrS}}_{2}$ layer separated from a metal layer by a nanoscale spacer, where t and g denote the ${\mathrm{ZrS}}_{2}$ thickness and the ${\mathrm{ZrS}}_{2}$–metal separation, respectively. (e) Calculated near‐field distributions of representative ${\mathrm{ZrS}}_{2}$ phonon–polariton modes at 250 ${\mathrm{cm}}^{-1}$ in RB1. (f) Calculated phonon–polariton dispersion of ${\mathrm{ZrS}}_{2}$ for a representative thickness of t=150 nm and g=5 nm, with polaritonic branches labeled by the branch index n.

The 1T crystal structure of ${\mathrm{ZrS}}_{2}$, consistent with that of other group‐IVB TMDs, gives rise to a broad Reststrahlen response and the associated hyperbolic dispersion. The in‐plane ($\varepsilon_{∥}$) and out‐of‐plane ($\varepsilon_{⊥}$) components of the complex permittivity are presented in Figure 1a, extracted using a Lorentz‐oscillator model [28]. For the in‐plane direction, the transverse and longitudinal optical phonons are located at 181 ${\mathrm{cm}}^{-1}$ ($\omega_{\mathrm{TO},∥}$) and 351 ${\mathrm{cm}}^{-1}$ ($\omega_{\mathrm{LO},∥}$), respectively, whereas in the out‐of‐plane direction, the corresponding phonons appear at 346 ${\mathrm{cm}}^{-1}$ ($\omega_{\mathrm{TO},⊥}$) and 368 ${\mathrm{cm}}^{-1}$ ($\omega_{\mathrm{LO},⊥}$). Here, RB1 denotes the in‐plane Reststrahlen band between $\omega_{\mathrm{TO},∥}$ and $\omega_{\mathrm{LO},∥}$, whereas RB2 denotes the out‐of‐plane Reststrahlen band between $\omega_{\mathrm{TO},⊥}$ and $\omega_{\mathrm{LO},⊥}$. In the type‐II hyperbolic portion of RB1, where Re(ε∥)<0 and Re(ε⊥)>0, the isofrequency contour becomes hyperbolic, as illustrated in Figure 1b. At the onset of RB2, where RB1 and RB2 overlap, both $\varepsilon_{∥}$ and $\varepsilon_{⊥}$ can have negative real parts, leading to a non‐hyperbolic double‐negative response rather than type‐I hyperbolicity. Type‐I hyperbolicity appears only in the non‐overlapping portion of RB2, where Re(ε∥)>0 while Re(ε⊥)<0, as shown by the representative isofrequency contour in Figure 1c. Note that the precise spectral extent and hyperbolic assignment of RB2 depend on the adopted out‐of‐plane Lorentz parameters, which are less consistently established in the literature than the in‐plane parameters [28, 29, 30, 48, 49]. Therefore, RB2 is treated as a representative high‐frequency Reststrahlen feature, whereas the main polariton analysis is based on the better‐constrained RB1 response.

Using the permittivity data in Figure 1a, we numerically investigated the dispersion and near‐field characteristics of HPhPs in ${\mathrm{ZrS}}_{2}$ (see Note S1 and Figure S1 for the dispersion derivation). The model geometry (Figure 1d) consists of a ${\mathrm{ZrS}}_{2}$ layer separated from a metallic substrate by a few‐nanometer‐thick dielectric spacer, replicating the experimental configuration used in this work to excite HPhPs through far‐field coupling to metal nanostructures. The metallic layer generates a mirror image of the polaritonic charge distribution in ${\mathrm{ZrS}}_{2}$, thereby increasing the in‐plane momentum of the HPhP relative to the freestanding case through a mechanism analogous to previously reported image phonon polaritons [11] (see Figures S2 and S3 for the gap‐size dependence). However, the momentum increase remains modest owing to the relatively large flake thickness (≈150 nm) considered here (see Figure S4 for the effect of ${\mathrm{ZrS}}_{2}$ thickness). In this relatively thick‐flake geometry, the dielectric spacer primarily serves to prevent possible interfacial damage during metal deposition and to reduce additional metal‐induced damping arising from direct ${\mathrm{ZrS}}_{2}$–metal contact, rather than to substantially enhance the polariton momentum.

The near‐field distribution of the phonon polariton at 250 ${\mathrm{cm}}^{-1}$ (Figure 1e) clearly demonstrates its hyperbolic propagation pattern [1, 8]. The corresponding dispersion relation for the same geometry (Figure 1f) reveals multiple polaritonic branches, labeled by the branch index n, within the frequency range between $\omega_{\mathrm{TO},∥}$ and $\omega_{\mathrm{TO},⊥}$, which arise from the finite thickness of the ${\mathrm{ZrS}}_{2}$ slab. The fundamental mode carries the strongest spectral weight, which progressively diminishes for higher‐order branches. In the RB2 region, the calculated upper branch exhibits a negative slope, corresponding to a negative group velocity. This negative‐slope dispersion is consistent with the characteristic behavior previously reported for type‐I hyperbolic HPhPs [5, 50]. However, owing to the relatively narrow RB2 bandwidth and larger damping, this branch is expected to exhibit weak far‐field spectral weight.

### 2.2 Far‐Field Observation of ${\mathrm{ZrS}}_{2}$ Phonon Polaritons

In contrast to scattering‐type near‐field optical microscopy (SNOM) [1, 4, 32, 51], which is commonly used to characterize HPhPs in various polar materials, we fabricated a ${\mathrm{ZrS}}_{2}$ phonon–polariton resonator that can be probed using a far‐field measurement setup such as a Fourier‐transform infrared (FTIR) spectrometer. Compared with SNOM, this far‐field approach enables broadband spectral analysis over large areas without the need for complex interferometric alignment [23, 52, 53].

Efficient far‐field excitation of ${\mathrm{ZrS}}_{2}$ phonon polaritons is achieved using a metal–spacer resonator architecture designed to suppress scattering losses. Several resonator‐assisted approaches have been developed to couple far‐field radiation to highly confined polaritonic modes. Conventional approaches often rely on directly patterning the polar material into nanoresonators [52], whereas antenna‐mediated coupling using nanometric metal particles has also been used to excite highly confined graphene plasmons and phonon polaritons in unpatterned polariton‐supporting materials [54, 55].

**FIGURE 2:**
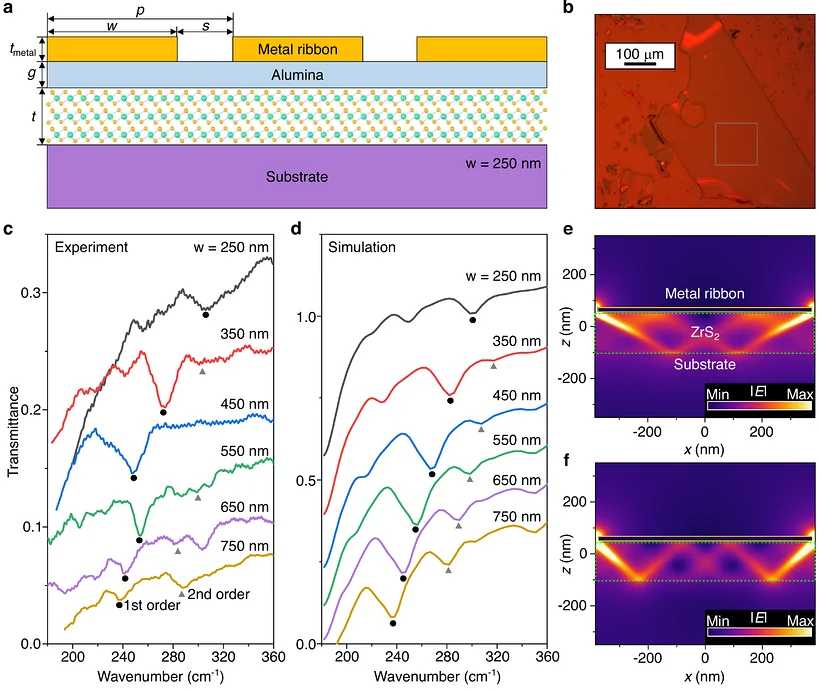
Far‐field observation of HPhPs in ${\mathrm{ZrS}}_{2}$. (a) Schematic of a ${\mathrm{ZrS}}_{2}$ phonon–polariton resonator. Here, p, w, and $t_{\mathrm{metal}}$ denote the periodicity, width, and thickness of the metal ribbons, respectively, while g and t represent the physical gap between the metal ribbon array and the ${\mathrm{ZrS}}_{2}$ layer and the thickness of the ${\mathrm{ZrS}}_{2}$ layer. (b) Optical microscope image of a ${\mathrm{ZrS}}_{2}$ flake. (c) Experimentally measured and (d) numerically calculated far‐infrared spectra of ${\mathrm{ZrS}}_{2}$ resonators for ribbon widths ranging from 250 to 750 nm. Calculated near‐field distributions of (e) the first‐order and (f) the second‐order phonon polariton resonances for w=750 nm.

The resonator design used in this work is illustrated in Figure 2a. In contrast to directly patterned polaritonic resonators, our design employs an unpatterned ${\mathrm{ZrS}}_{2}$ flake coupled to a metal ribbon array through a few‐nanometer‐thick alumina spacer. To enhance the coupling efficiency, the spacing between adjacent metal ribbons, s, is reduced to 20 nm. The metal and spacer thicknesses are fixed at 30 and 5 nm, respectively. This resonator geometry enables efficient far‐field coupling to propagating HPhPs while minimizing edge‐related scattering and surface‐roughness‐induced losses in the ${\mathrm{ZrS}}_{2}$ layer [11, 56]. Optical micrographs in Figure 2b show exfoliated flakes approximately 140 nm thick and 170 μm wide, sufficiently large to match the ∼100 μm FTIR beam spot.

The FTIR‐measured transmission spectra for different ribbon widths w are shown in Figure 2c. Each spectrum exhibits multiple absorption dips with one dominant resonance that generally redshifts as w increases. This trend is consistent with the numerically simulated spectra in Figure 2d, which predict both a strong fundamental resonance and a weaker second‐order mode. Although the second‐order resonance is less pronounced experimentally, it can still be identified in the measured spectra (solid triangle symbols in Figure 2c). The weaker intensity of this second‐order resonance in the experiment compared with the simulation can be attributed to fabrication‐induced geometrical variations, such as deviations in ribbon width or period, as well as local variations in ${\mathrm{ZrS}}_{2}$ thickness, crystal quality, and dielectric response. These nonidealities may also contribute to the slight nonmonotonic variation of the measured resonance frequency with ribbon width. Simulated near‐field distributions for the first‐ and second‐order resonances for w=750 nm are shown in Figure 2e,f. Both resonances exhibit characteristic HPhP field patterns, in which the hyperbolic rays launched at the ribbon edges undergo multiple internal reflections within the ${\mathrm{ZrS}}_{2}$ slab. Due to the larger incident angle of the hyperbolic ray with respect to the in‐plane direction, the second‐order resonance shows additional field oscillations across the ribbon period.

### 2.3 Far‐Field Excitation of Higher‐Order Polaritonic Branches

**FIGURE 3:**
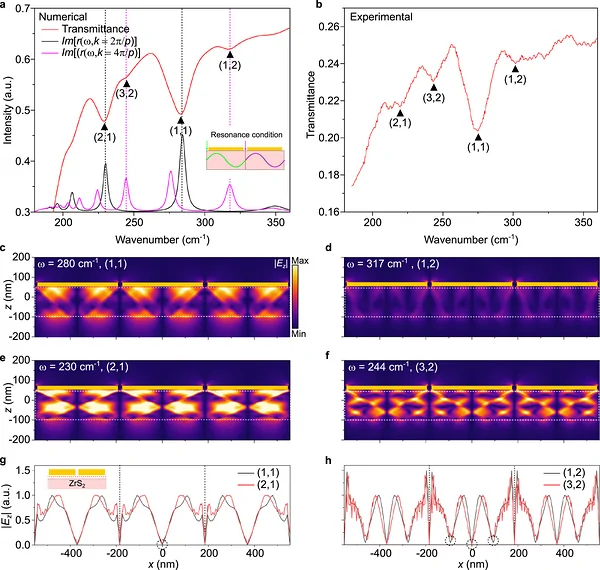
Higher‐order resonances arising from multiple polaritonic branches in ${\mathrm{ZrS}}_{2}$. (a) Full‐wave‐simulated transmittance spectrum of the ${\mathrm{ZrS}}_{2}$ resonator for w=350 nm, together with calculated loss spectra evaluated at representative momenta k=2π/p and k=4π/p, corresponding to the first (m=1) and second (m=2) momentum orders, respectively. The labeled resonances are denoted as (n,m), where n indicates the polaritonic branch index and m indicates the ribbon–array momentum order. (b) Experimental FTIR transmittance spectrum of the ${\mathrm{ZrS}}_{2}$ resonator for w=350 nm. (c–f) Calculated near‐field distributions of representative resonances labeled in (a): (c) (1,1) at 280 ${\mathrm{cm}}^{-1}$, (d) (1,2) at 317 ${\mathrm{cm}}^{-1}$, (e) (2,1) at 230 ${\mathrm{cm}}^{-1}$, and (f) (3,2) at 244 ${\mathrm{cm}}^{-1}$. (g,h) Line profiles of $|E_{z}|$ in the gap region between the metal ribbons and the ${\mathrm{ZrS}}_{2}$ layer. The inset in (g) shows the line‐cut position indicated by the red line, and the dotted circles in (g,h) mark representative field minima.

In addition to the fundamental and second‐order resonances discussed above, the metal‐ribbon resonator enables far‐field access to several higher‐order polaritonic branches. In this resonator design, the resonance condition can be expressed as $k_{n}\left(\omega \right)\approx mG=2\pi m/p$, where $k_{n}\left(\omega \right)$ is the in‐plane momentum of the nth polaritonic branch, G=2π/p is the reciprocal lattice vector of the ribbon array and m is an integer denoting the ribbon‐array momentum order. Figure 3a shows the full‐wave‐simulated transmittance spectrum of the complete device stack for w=350 nm, s=20 nm, and g=5 nm together with the calculated loss spectra evaluated at representative momentum orders of m=1 and m=2. The multiple peaks in each loss spectrum do not represent different momentum orders; rather, they correspond to different polaritonic branches in the dispersion. We therefore label each resonance as (n,m). In each loss spectrum, the first branch corresponds to the highest frequency peak, and higher branch indices denote progressively lower‐frequency branches. The peak positions obtained from the loss spectra agree well with the full‐wave‐simulated transmittance spectrum, enabling assignment of the simulated resonances. Guided by the numerical results in Figure 3a, the resonances in the experimentally measured spectrum are assigned as shown in Figure 3b, confirming far‐field observation of resonances associated with multiple polaritonic branches and momentum orders.

The numerically calculated near‐field distributions corresponding to the observed resonances are shown in Figure 3c–f. In all cases, the fields inside the ${\mathrm{ZrS}}_{2}$ layer consist of hyperbolic rays launched from the apertures between adjacent metal ribbons. These ray patterns are governed primarily by the hyperbolic ray angle θ, which is set by the anisotropic permittivity tensor at a given frequency according to $\tan\theta \approx \sqrt{|\varepsilon_{⊥}/\varepsilon_{∥}|}$, together with the thickness of the ${\mathrm{ZrS}}_{2}$ slab. To identify the resonance order more directly, we examine the z component of the electric field in the gap region between the metal ribbons and the ${\mathrm{ZrS}}_{2}$ layer, as shown in Figure 3g,h. The first‐order resonances, (1,1) and (2,1), exhibit a similar gap‐field profile with a single field minimum near the center of the unit cell. By contrast, the second‐order resonances, (1,2) and (3,2), show three field minima within the unit cell. The increase in the number of field minima with increasing momentum order is also observed for higher‐order resonances (see Figure S5). These results show that the gap‐region field profile reflects the momentum order, whereas the hyperbolic ray trajectory inside ${\mathrm{ZrS}}_{2}$ is mainly controlled by the frequency‐dependent permittivity tensor.

### 2.4 Dispersion and Intrinsic Light–Matter Coupling Strength

**FIGURE 4:**
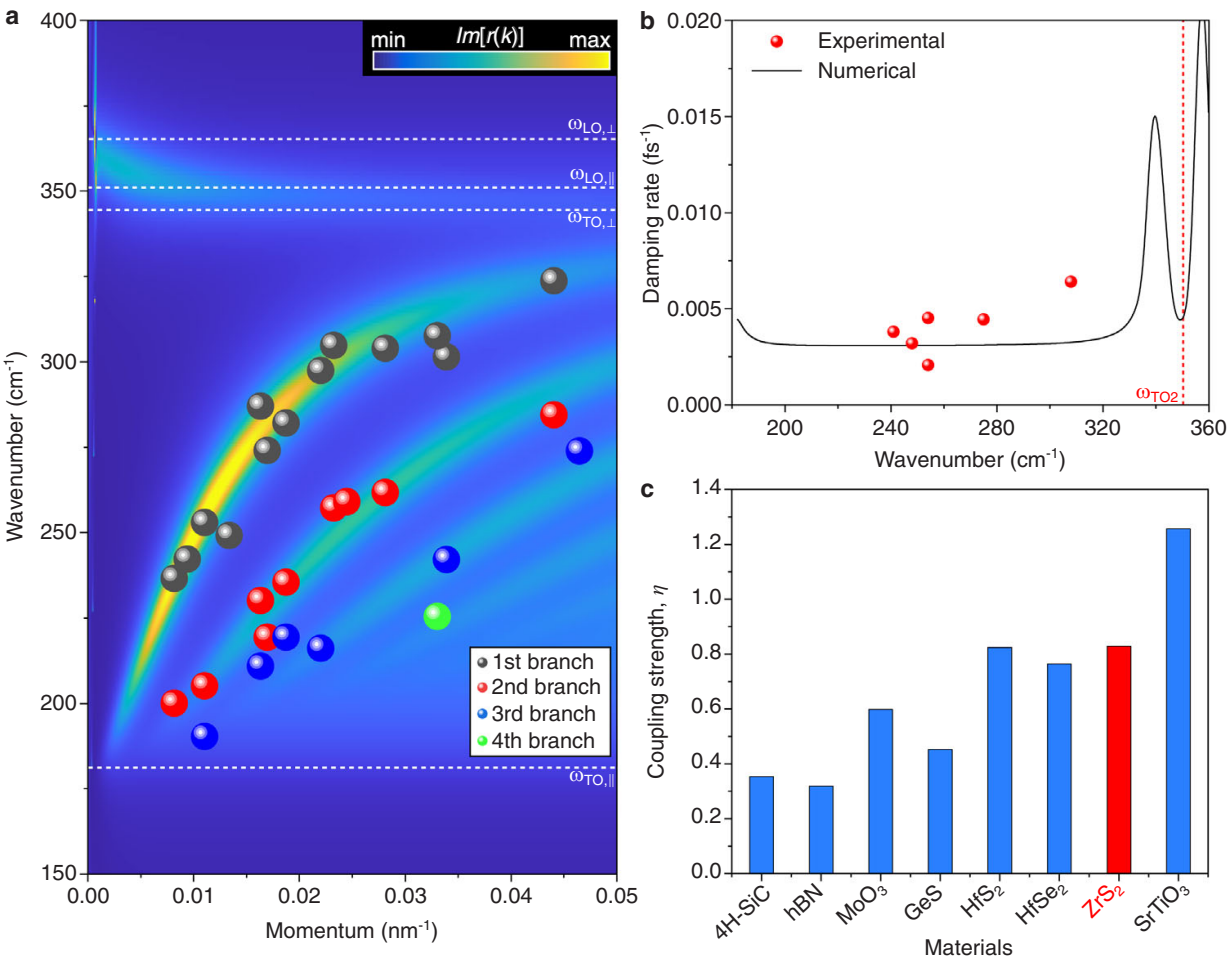
Dispersion and intrinsic light–matter coupling strength. (a) Numerically calculated dispersion of ${\mathrm{ZrS}}_{2}$ phonon polaritons overlaid with experimentally determined dispersion. (b) Experimentally extracted polariton damping rates compared with theoretical calculations. (c) Normalized light–matter coupling strengths of representative phonon–polariton–supporting materials [32, 57].

From the identified resonances, we extracted the in‐plane momenta of the phonon polaritons and compared them with the numerical results shown in Figure 4a. The experimentally obtained data points align well with the simulated dispersion and can be assigned to multiple phonon‐polariton branches, extending up to the fourth branch (see Figures S6–S8 for resonance assignment). The results reveal an extremely confined mode, with an effective refractive index ($k_{p}/k_{0}$) as high as 223, estimated from the (3,2) resonance in Figure 3b. This level of confinement approaches the highest values reported in group‐IVB TMDs [32], where ${\mathrm{HfSe}}_{2}$ demonstrated confinement factors of 250 on silicon and 95 on ${\mathrm{SiO}}_{2}$, underscoring the exceptional potential of 1T TMDs as a material family for extreme far‐field phonon–polariton confinement. Additional weak features at higher momenta are discussed in the Supporting Information, but they are not used for the primary quantitative analysis because of their weaker spectral contrast.

The damping rates were extracted from the linewidths of the first‐order resonances [11, 58] and compared with the analytical results shown in Figure 4b (see Note S2 and Figure S9 for damping analysis). The analytical loss model considers only the intrinsic propagation loss of ${\mathrm{ZrS}}_{2}$ phonon–polaritons and neglects extrinsic contributions arising from surface roughness and edge scattering associated with the metal ribbons. The good agreement between experiment and theory indicates that the measured linewidth is primarily governed by intrinsic polariton propagation loss, with extrinsic contributions minimized by the unpatterned ${\mathrm{ZrS}}_{2}$/metal‐ribbon resonator design, similar to previously reported resonator architectures based on unpatterned two‐dimensional materials [11, 56]. The minimum damping rate obtained here is $\gamma =2.07\times 10^{12}\;s^{-1}$, corresponding to a linewidth‐derived lifetime of τ≈0.48 ps using τ=1/γ. For broader context, propagating phonon polaritons in benchmark systems such as hBN [10], α‐${\mathrm{MoO}}_{3}$ [51], and ${\mathrm{YVO}}_{4}$ [20] can exhibit reported lifetimes of a few picoseconds, with many of these studies performed in the mid‐infrared regime (see Table S1 for benchmarking). The shorter lifetime observed in ${\mathrm{ZrS}}_{2}$ is consistent with its relatively large intrinsic optical‐phonon broadening, which may arise from stronger thermally assisted anharmonic scattering of low‐energy far‐IR phonons [59].

${\mathrm{ZrS}}_{2}$ exhibits strong light–matter coupling in the far‐IR regime. This interaction is quantified using the normalized coupling strength, $\eta =g_{c}/\omega_{\mathrm{TO}}=\sqrt{\omega_{\mathrm{LO}}^{2}-\omega_{\mathrm{TO}}^{2}}/\left(2\omega_{\mathrm{TO}}\right)$, where $g_{c}$ is the coupling strength [32]. As shown in Figure 4c, η for ${\mathrm{ZrS}}_{2}$ reaches 0.83, placing it among the largest values reported for phonon–polaritonic materials to date. This value exceeds that of ${\mathrm{HfS}}_{2}$ and is comparable to that of ${\mathrm{HfSe}}_{2}$, two related group‐IVB TMDs [32]. Similarly large values have been reported for ${\mathrm{SrTiO}}_{3}$ [60, 61], an epitaxially grown oxide thin film rather than a van der Waals material. This large normalized coupling strength of ${\mathrm{ZrS}}_{2}$ reflects an enhanced oscillator strength arising from the combination of large Born effective charges and a relatively low transverse optical phonon frequency.

## 3 Conclusion

In conclusion, we have experimentally demonstrated far‐IR HPhPs in the group‐IVB TMD ${\mathrm{ZrS}}_{2}$ using a resonator‐assisted far‐field spectroscopy platform. The resonator design based on an unpatterned ${\mathrm{ZrS}}_{2}$ flake enables efficient far‐field excitation of phonon polaritons while suppressing extrinsic scattering and surface‐roughness‐induced losses. As a result, we observe multiple polaritonic resonances beyond the fundamental branch. The experimentally determined dispersion reveals ultrahigh in‐plane wavevectors corresponding to an effective refractive index as large as 223, while linewidth analysis confirms that polariton damping is dominated by intrinsic propagation loss. This extreme polariton confinement is enabled by the large light–matter coupling strength of ${\mathrm{ZrS}}_{2}$, arising from its strong phonon oscillator strength and low transverse optical phonon frequency.

These results establish ${\mathrm{ZrS}}_{2}$ as a new van der Waals hyperbolic material platform capable of supporting low‐damping, ultraconfined phonon polaritons in the far‐IR regime. More broadly, this work highlights the untapped potential of group‐IVB TMDs for far‐infrared polaritonics and provides a general framework for accessing HPhPs through scalable far‐field architectures. By enabling deeply subwavelength control of low‐energy infrared excitations, our findings open new opportunities in compact far‐IR polaritonic devices, including ultra‐compact waveguides [38], polarization turners [39], canalization lenses [62], and on‐chip steering architectures [40], where the ultrahigh in‐plane momentum of ${\mathrm{ZrS}}_{2}$ HPhPs can enable dense integration and compact beam control. They also point toward applications in surface‐enhanced infrared spectroscopy [36, 37] and spectrally selective thermal nanophotonics [34, 35], while bridging the spectral gap between mid‐infrared and terahertz regimes.

## 4 Experimental Section

### 4.1 Device Fabrication

${\mathrm{ZrS}}_{2}$ single‐crystal flakes were mechanically exfoliated from bulk crystals (2D Semiconductors Inc.) onto double‐side‐polished Si substrates (thickness: 500 μm) using a standard dry‐transfer method [63]. The resulting flakes had thicknesses ranging from 140 to 170 nm and typical lateral dimensions exceeding 150 μm. All exfoliation, handling, and storage procedures were carried out in an inert‐atmosphere glovebox to prevent oxidation.

To define the spacer layer and promote uniform atomic layer deposition (ALD), a 1 nm Al seed layer was first deposited by electron‐beam evaporation (CHA SEC 600) and subsequently oxidized to form ${\mathrm{Al}}_{2}O_{3}$. A conformal ${\mathrm{Al}}_{2}O_{3}$ spacer layer with a thickness of 5 nm was then deposited by ALD (Cambridge NanoTech). Gold ribbon arrays (30 nm Au with a 1 nm Ti adhesion layer) were patterned using electron‐beam lithography (Vistec EBPG 5000+) with 950 PMMA C2 resist spun at 4000 rpm for 30 s. Following development in MIBK:IPA (1:3) for 60 s, the Au/Ti layers were patterned by ${\mathrm{Ar}}^{+}$ ion milling (Intlvac Nanoquest).

### 4.2 Optical Characterization

Far‐infrared transmission spectra were acquired using a Bruker Hyperion 2000 infrared microscope coupled to a Bruker Vertex 70v FTIR spectrometer. A 36× Cassegrain objective was employed, and the transmitted signal was detected using a closed‐cycle superconducting bolometer (QMC Technologies). All measurements were performed under vacuum conditions with a spectral resolution of 4 ${\mathrm{cm}}^{-1}$.

### 4.3 Numerical Simulations

Numerical simulations were performed using finite‐element analysis in COMSOL Multiphysics. The anisotropic dielectric response of ${\mathrm{ZrS}}_{2}$ was modeled using the Lorentz‐oscillator parameters described in Note S1. The model consisted of periodic Au ribbon arrays positioned above a ${\mathrm{ZrS}}_{2}$ slab and separated by an ${\mathrm{Al}}_{2}O_{3}$ spacer layer with a thickness of 5 nm. The spacing between the metal ribbons was fixed at 20 nm. Periodic boundary conditions were applied along the in‐plane directions, and perfectly matched layers were used to absorb outgoing waves.

## Conflicts of Interest

The authors declare no conflicts of interest.

## Supporting information


**Supporting File**: adma74154‐sup‐0001‐SuppMat.pdf.

## Data Availability

The data that support the findings of this study are available from the corresponding author upon reasonable request.
