# Supplementary material for: Far‐Infrared Hyperbolic Phonon–Polaritons in Zirconium Disulfide
Source: Adv Mater. 2026 Aug 6;38(52):e74154. doi: 10.1002/adma.74154 (PMC13579202; doi:10.1002/adma.74154)
Supplement: Supplementary file 1 [file ADMA-38-e74154-s001.pdf]

Supporting Information: Far-infrared hyperbolic phonon-polaritons in Zirconium Disulfide

Subhodip Saha[1], Ryan Kowalski[2,3], Joseph R. Matson[2,3], Thomas G. Folland[4], Tony Low[1], Joshua D. Caldwell[2,3], In-Ho Lee[5,6,*], Sang-Hyun Oh[1,*]

[1]Department of Electrical and Computer Engineering, University of Minnesota, Minneapolis, MN 55455, USA

[2]Department of Mechanical Engineering, Vanderbilt University, Nashville, TN 37235, USA

[3]Interdisciplinary Materials Science Program, Vanderbilt University, Nashville, TN 37212, USA

[4]Department of Physics and Astronomy, The University of Iowa, Iowa City, IA 52242, USA

[5]Center for Quantum Technology, Post-Silicon Semiconductor Institute, Korea Institute of Science and Technology (KIST), Seoul 02792, Republic of Korea

[6]Division of Nanoscience and Technology, KIST School at University of Science and Technology, Seoul, Republic of Korea, Seoul 02792, Republic of Korea

***Corresponding authors:** inholee87@kist.re.kr, sang@umn.edu

Supplementary Note 1. Phonon–polariton dispersion in ZrS_2

We consider a three-layer geometry consisting of medium I ($y < 0$), medium II ($0 < y < t$), and medium III ($t < y < g$), as illustrated in Fig. S1a. Medium II is a uniaxial van der Waals polar crystal, zirconium disulfide (ZrS_2), while media I and III are isotropic dielectrics. A perfect electric conductor is placed at $y = g$.

The magnetic fields $\mathbf{H}(x,y,z)$ in the three regions are written as

$$\mathbf{H}_l(x,y,z) = \begin{cases} \hat{\mathbf{e}}_z \left(H_\mathrm{i} e^{ik_{y\mathrm{I}}y} + H_\mathrm{r} e^{-ik_{y\mathrm{I}}y}\right) e^{ikx}, & l = \mathrm{I}, \\ \hat{\mathbf{e}}_z \left(H_{2\mathrm{p}} e^{ik_{y\mathrm{II}}y} + H_{2\mathrm{n}} e^{-ik_{y\mathrm{II}}y}\right) e^{ikx}, & l = \mathrm{II}, \\ \hat{\mathbf{e}}_z \left(H_{3\mathrm{p}} e^{ik_{y\mathrm{III}}y} + H_{3\mathrm{n}} e^{-ik_{y\mathrm{III}}y}\right) e^{ikx}, & l = \mathrm{III}. \end{cases} \tag{S1}$$

Here k and k_{yl} denote the wavevector components along the x and y directions, respectively. The free-space wavenumber is $k_0 = \omega/c$. For the isotropic media I and III,

$$k_{yl} = \sqrt{\varepsilon_l k_0^2 - k^2}, \qquad l = \mathrm{I}, \mathrm{III}, \tag{S2}$$

where ε_l is the complex permittivity of medium l.

For the uniaxial ZrS_2 slab (medium II), characterized by the dielectric tensor $\varepsilon_2 = \mathrm{diag}(\varepsilon_{2\parallel}, \varepsilon_{2\parallel}, \varepsilon_{2\perp})$, the TM-polarized normal wavevector component is

$$k_{y\mathrm{II}} = \sqrt{\frac{\varepsilon_{2\parallel}}{\varepsilon_{2\perp}} k^2 - \varepsilon_{2\parallel} k_0^2}. \tag{S3}$$

The corresponding electric fields are obtained from Maxwell's equations as

$$\mathbf{E}_l(x,y,z) = \frac{1}{i\omega\varepsilon_l} \left(\hat{\mathbf{e}}_y\, ik \pm \hat{\mathbf{e}}_x\, ik_{yl}\right) H_l e^{\pm ik_{yl}y} e^{ikx}, \tag{S4}$$

where the upper (lower) sign corresponds to forward (backward) propagation along y.

The continuity of the tangential field components at the interfaces yields

At $y = 0$:

$$\begin{cases} H_\mathrm{i} + H_\mathrm{r} = H_{2\mathrm{p}} + H_{2\mathrm{n}}, \\ H_\mathrm{i} - H_\mathrm{r} = \alpha_1 \left(H_{2\mathrm{p}} + H_{2\mathrm{n}}\right), \end{cases} \tag{S5}$$

At $y = t$:

$$\begin{cases} H_{2\mathrm{p}} e^{ik_{y\mathrm{II}}t} + H_{2\mathrm{n}} e^{-ik_{y\mathrm{II}}t} = H_{3\mathrm{p}} e^{ik_{y\mathrm{III}}t} + H_{3\mathrm{n}} e^{-ik_{y\mathrm{III}}t}, \\ H_{2\mathrm{p}} e^{ik_{y\mathrm{II}}t} - H_{2\mathrm{n}} e^{-ik_{y\mathrm{II}}t} = \alpha_2 \left(H_{3\mathrm{p}} e^{ik_{y\mathrm{III}}t} + H_{3\mathrm{n}} e^{-ik_{y\mathrm{III}}t}\right), \end{cases} \tag{S6}$$

At $y = g$ (perfect conductor):

$$H_{3\mathrm{p}} e^{ik_{y\mathrm{III}}g} = H_{3\mathrm{n}} e^{-ik_{y\mathrm{III}}g}. \tag{S7}$$

The dimensionless interface parameters are defined as

$$\alpha_1 = \frac{k_{y\mathrm{II}}\varepsilon_1}{k_{y\mathrm{I}}\varepsilon_{2\parallel}}, \qquad \alpha_2 = \frac{k_{y\mathrm{III}}\varepsilon_{2\parallel}}{k_{y\mathrm{II}}\varepsilon_3}. \tag{S8}$$

From the boundary conditions, the TM reflection coefficient is obtained as

$$r_k = \frac{H_\mathrm{r}}{H_\mathrm{i}} = \frac{(1-\alpha_1)e^{-ik_{y\mathrm{II}}t}M + (1+\alpha_1)e^{ik_{y\mathrm{II}}t}N}{(1+\alpha_1)e^{-ik_{y\mathrm{II}}t}M + (1-\alpha_1)e^{ik_{y\mathrm{II}}t}N}, \tag{S9}$$

where

$$M = (1-\alpha_2)e^{ik_{y\mathrm{III}}(g-t)} + (1+\alpha_2), \tag{S10a}$$

$$N = (1+\alpha_2)e^{ik_{y\text{III}}(g-t)} + (1-\alpha_2). \tag{S10b}$$

The dispersion relation of the phonon polaritons supported by the ZrS_2 slab is determined by the pole condition of the reflection coefficient,

$$(1+\alpha_1)e^{-ik_{y\text{II}}t}M + (1-\alpha_1)e^{ik_{y\text{II}}t}N = 0. \tag{S11}$$

The anisotropic dielectric response of ZrS_2 is described using a Lorentz oscillator model. For a given wavelength λ (in μm), the permittivity is written as

$$\varepsilon_{2j}(\omega) = \varepsilon_{\infty,j}\left(1 + \frac{\omega_{\text{LO},j}^2 - \omega_{\text{TO},j}^2}{\omega_{\text{TO},j}^2 - \omega^2 - i\Gamma_j\omega}\right), \qquad j = \parallel, \perp, \tag{S12}$$

where $\omega = 10^4/\lambda$ is expressed in cm^{-1}. The parameters $\varepsilon_{\infty,j}$, $\omega_{\text{TO},j}$, $\omega_{\text{LO},j}$, and Γ_j are chosen separately for the in-plane ($j = \parallel$) and out-of-plane ($j = \perp$) directions.The complex refractive indices are obtained as $\tilde{n}_j = \sqrt{\varepsilon_{2j}}$, with the branch chosen to ensure a positive imaginary part. In this work, the anisotropic dielectric response of ZrS_2 is modeled using direction-dependent Lorentz-oscillator parameters. For the in-plane response, we use the experimentally reported optical phonon parameters for the E_u branch[1]. For the out-of-plane response, a complete experimental Lorentz-oscillator parameter set is not readily available; therefore, $\varepsilon_\perp$ is described using an effective Lorentz-oscillator model guided by reported A_{2u} phonon assignments and the high-frequency out-of-plane dielectric constant of bulk ZrS_2[2,3]. The parameters used in the calculations are specified below for the in-plane ($j = \parallel$) and out-of-plane ($j = \perp$) responses, with all phonon frequencies and damping constants given in cm^{-1}:

$$\text{In-plane } (j = \parallel): \quad \varepsilon_{\infty,\parallel} = 9.2,\ \omega_{\text{TO},\parallel} = 181,\ \omega_{\text{LO},\parallel} = 351,\ \Gamma_\parallel = 16.3, \tag{S13a}$$

$$\text{Out-of-plane } (j = \perp): \quad \varepsilon_{\infty,\perp} = 6.2,\ \omega_{\text{TO},\perp} = 346,\ \omega_{\text{LO},\perp} = 368,\ \Gamma_\perp = 16.3. \tag{S13b}$$

Here, $\Gamma_\perp$ is treated as an effective linewidth parameter and is set equal to the reported in-plane damping constant to avoid overestimating the out-of-plane loss in the absence of a complete experimental out-of-plane damping parameter. The spectral variable is $\omega = 10^4/\lambda$ in cm^{-1}, where λ is given in μm.

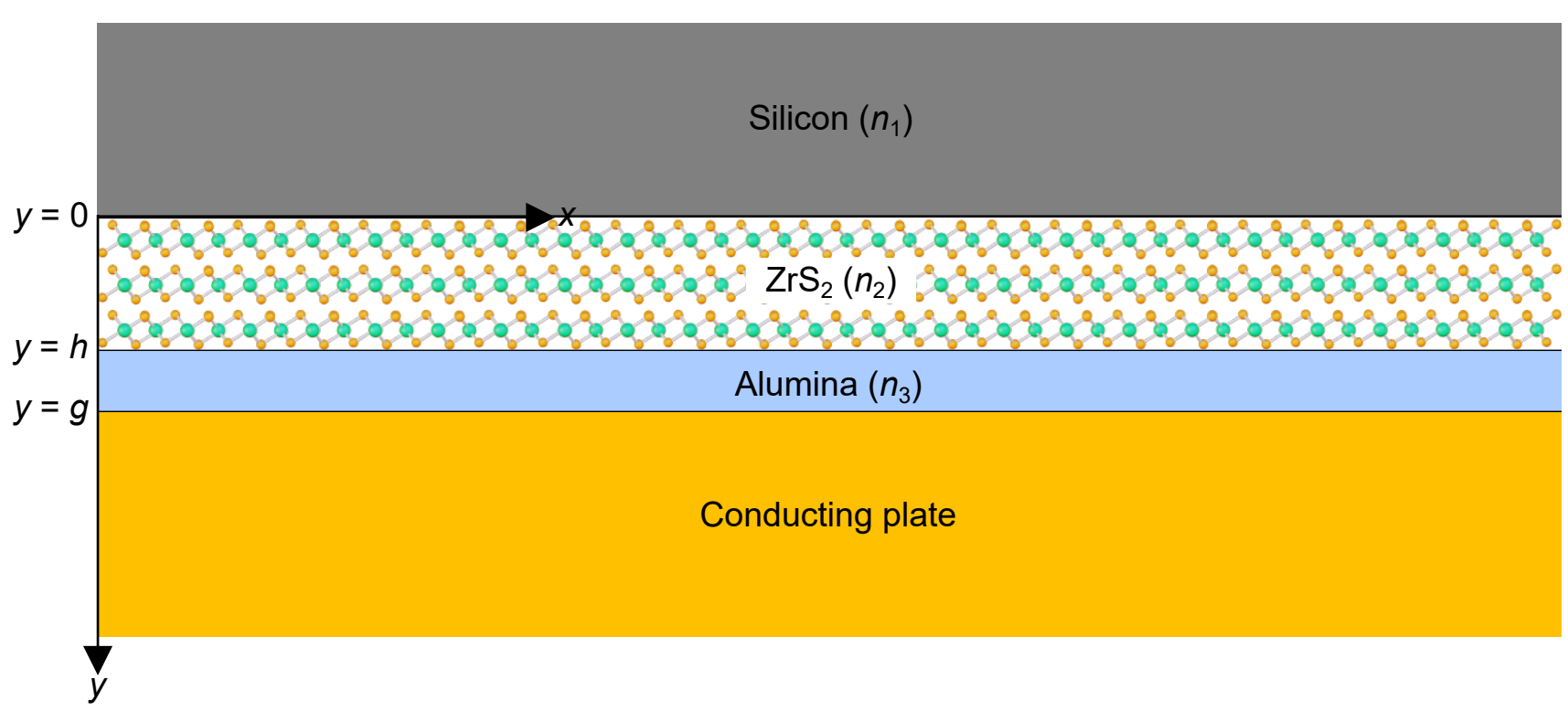

Figure S1. Schematic illustration of geometry of supporting far-infrared phonon polaritons.

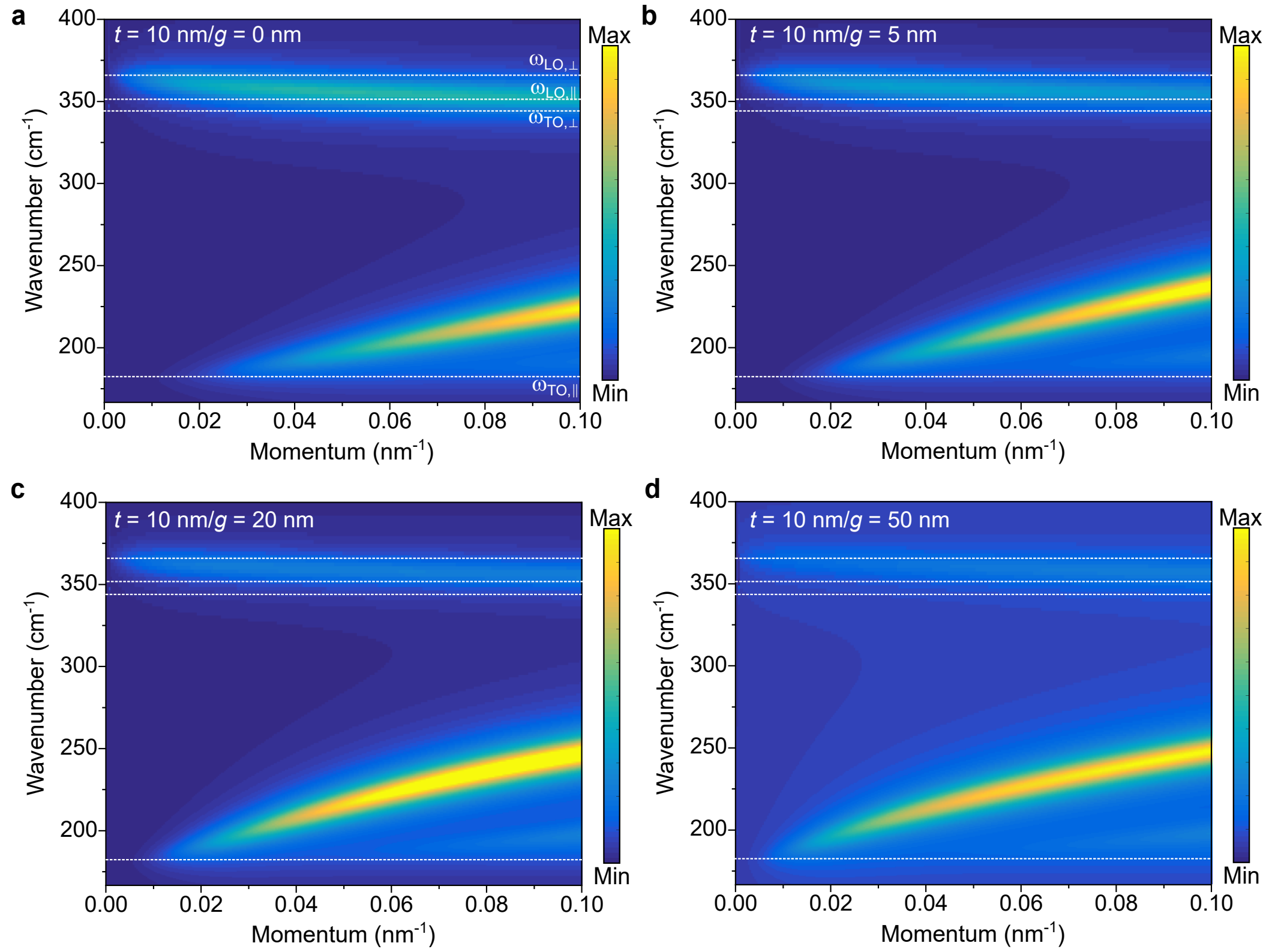

Figure S2. Gap-size dependence of ZrS_2 phonon–polariton dispersion. Calculated phonon–polariton dispersion relations of a ZrS_2 slab separated from a perfectly conducting plate by a gap of (a) $g = 0$ nm, (b) $g = 5$ nm, (c) $g = 20$ nm, and (d) $g = 50$ nm, while the ZrS_2 thickness is fixed at $t = 10$ nm.

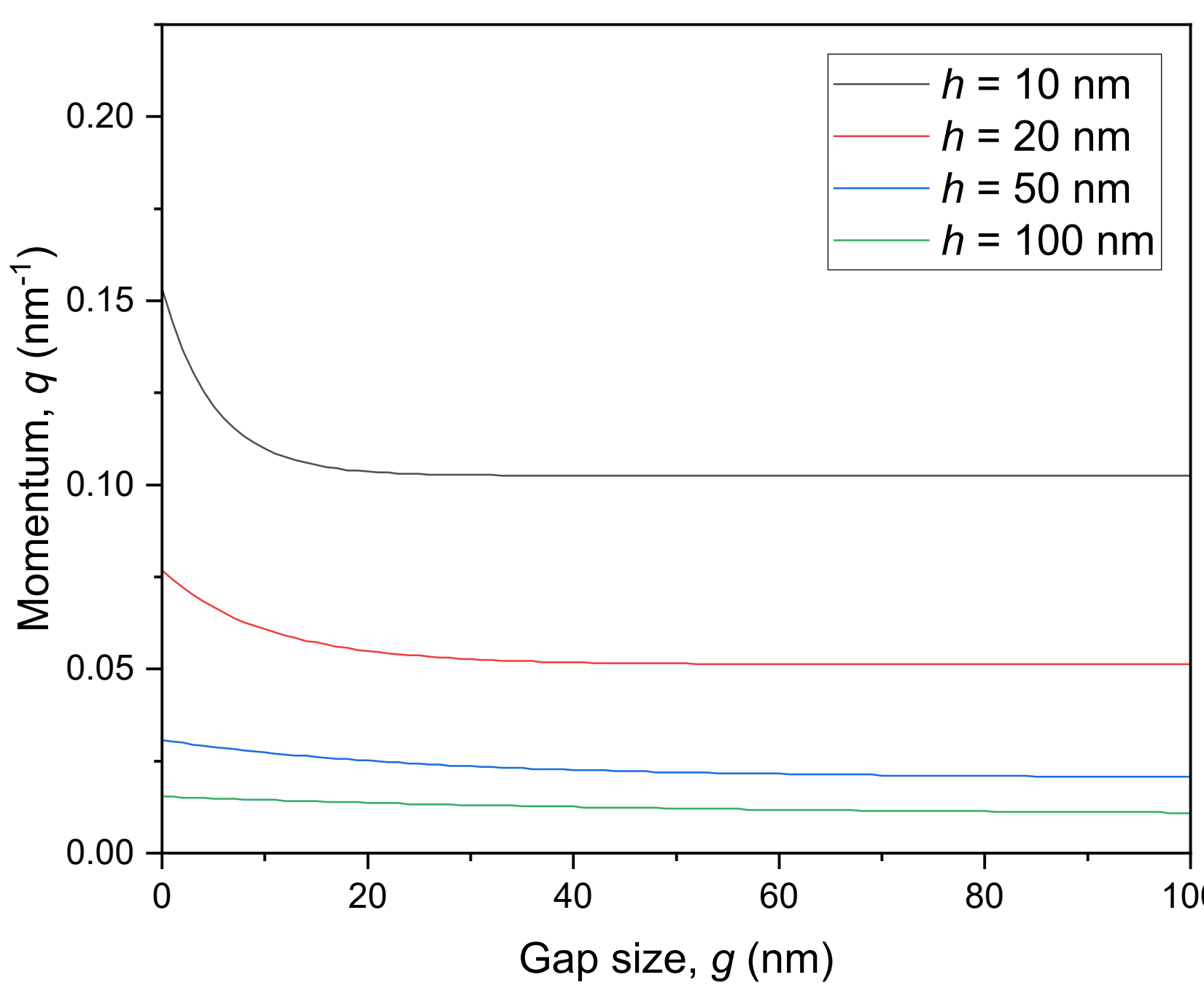

Figure S3. Gap-size dependence of phonon–polariton momentum in ZrS_2. Calculated phonon–polariton in-plane momenta of a ZrS_2 slab as a function of the gap size g for slab thicknesses ranging from $h = 10$ to 100 nm.

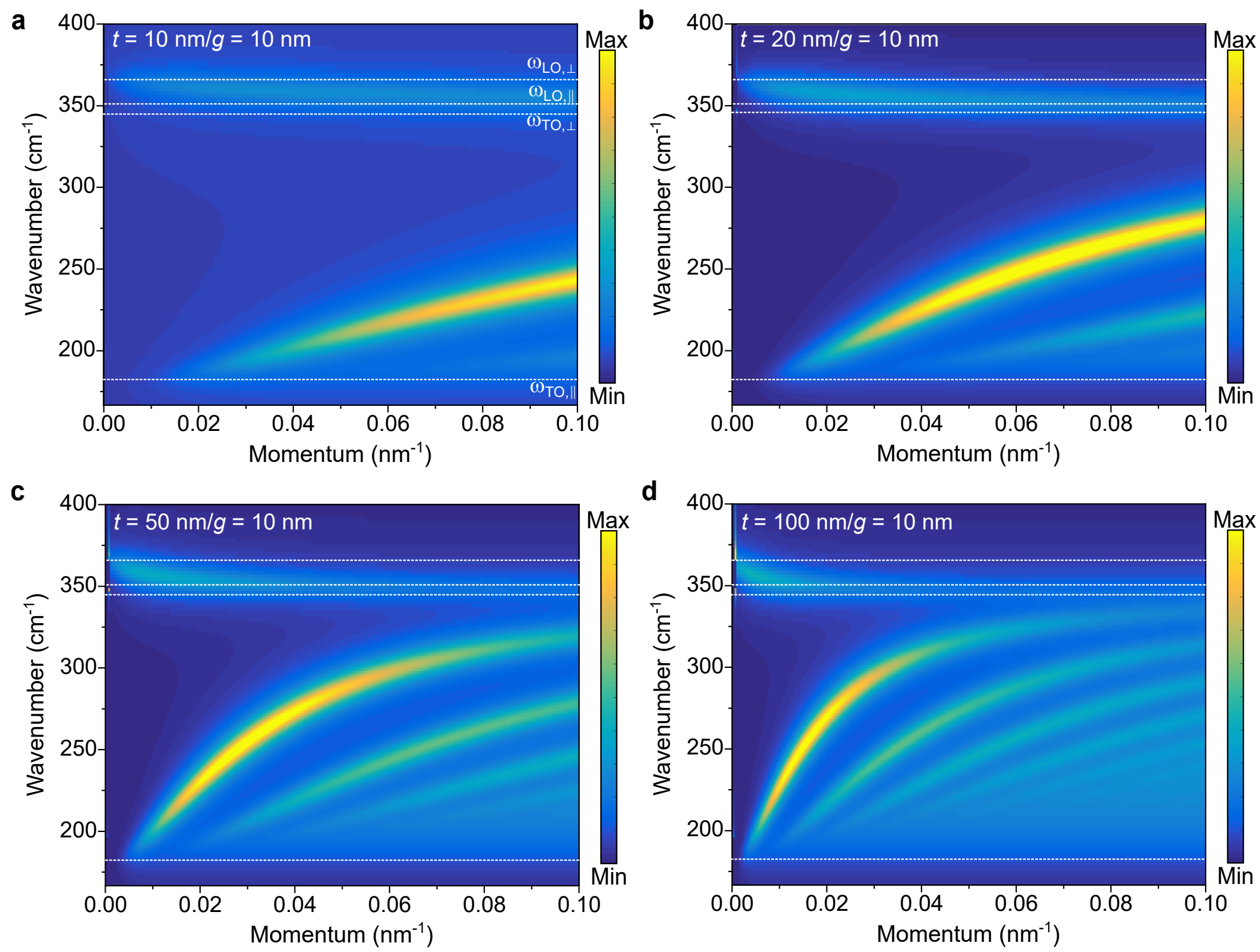

Figure S4. Thickness dependence of ZrS_2 phonon–polariton dispersion. Calculated phonon–polariton dispersion relations of a ZrS_2 slab with thickness (a) $t = 10$ nm, (b) $t = 20$ nm, (c) $t = 30$ nm, and (d) $t = 40$ nm, while the distance to the perfectly conducting plate is fixed at $g = 10$ nm.

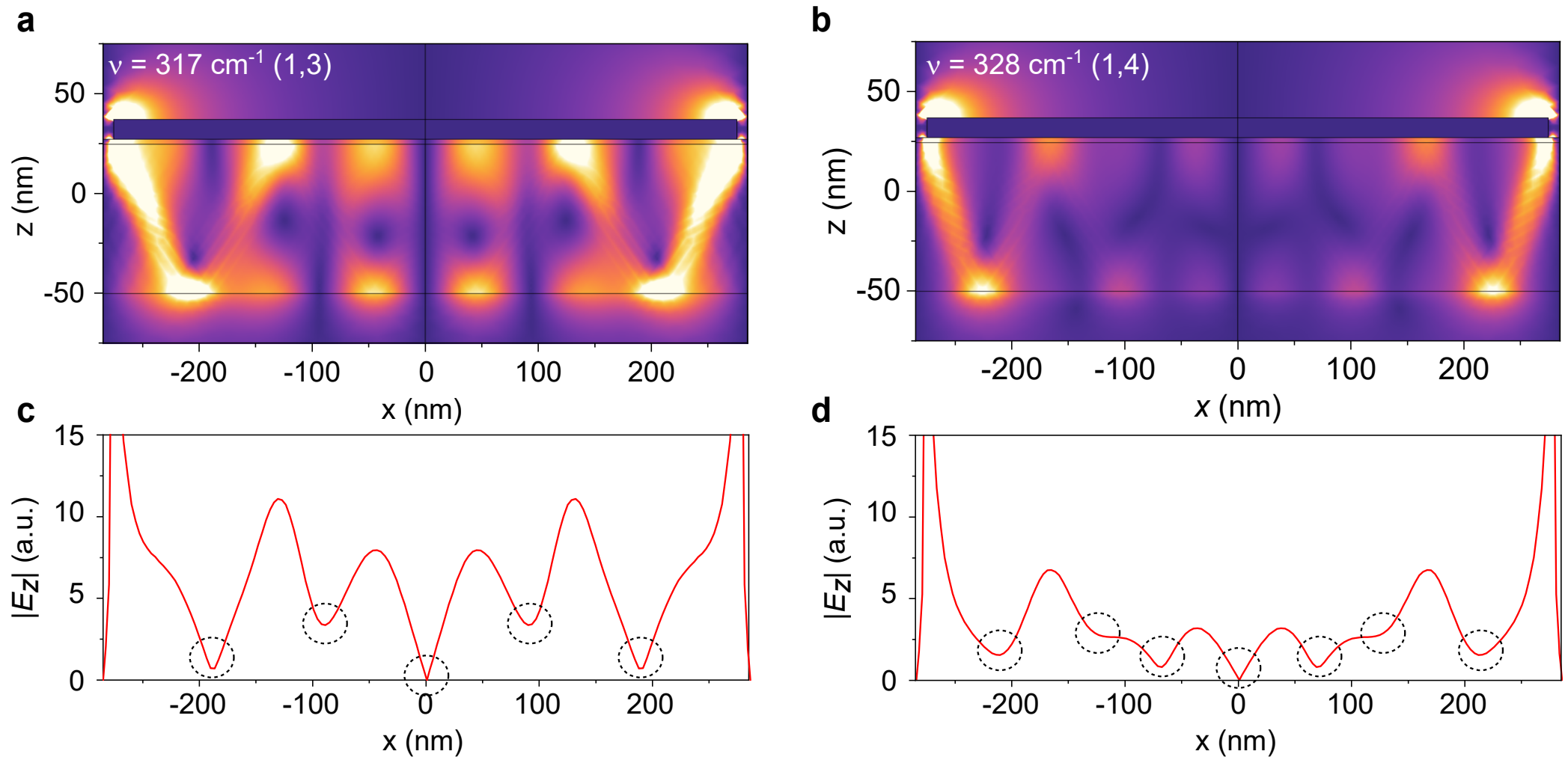

Figure S5. Near-field profiles of higher grating-order resonances. (a,b) Numerically calculated near-field distributions of representative higher grating-order resonances in the ZrS_2 resonator: (a) the third-order resonance, $(1,3)$, at 317 cm^{-1} and (b) the fourth-order resonance, $(1,4)$, at 328 cm^{-1}. (c,d) Corresponding line profiles of $|E_z|$ in the gap region between the metal ribbon array and the ZrS_2 layer for (c) $(1,3)$ and (d) $(1,4)$. The circled field minima indicate the characteristic spatial modulation associated with the higher grating momentum orders.

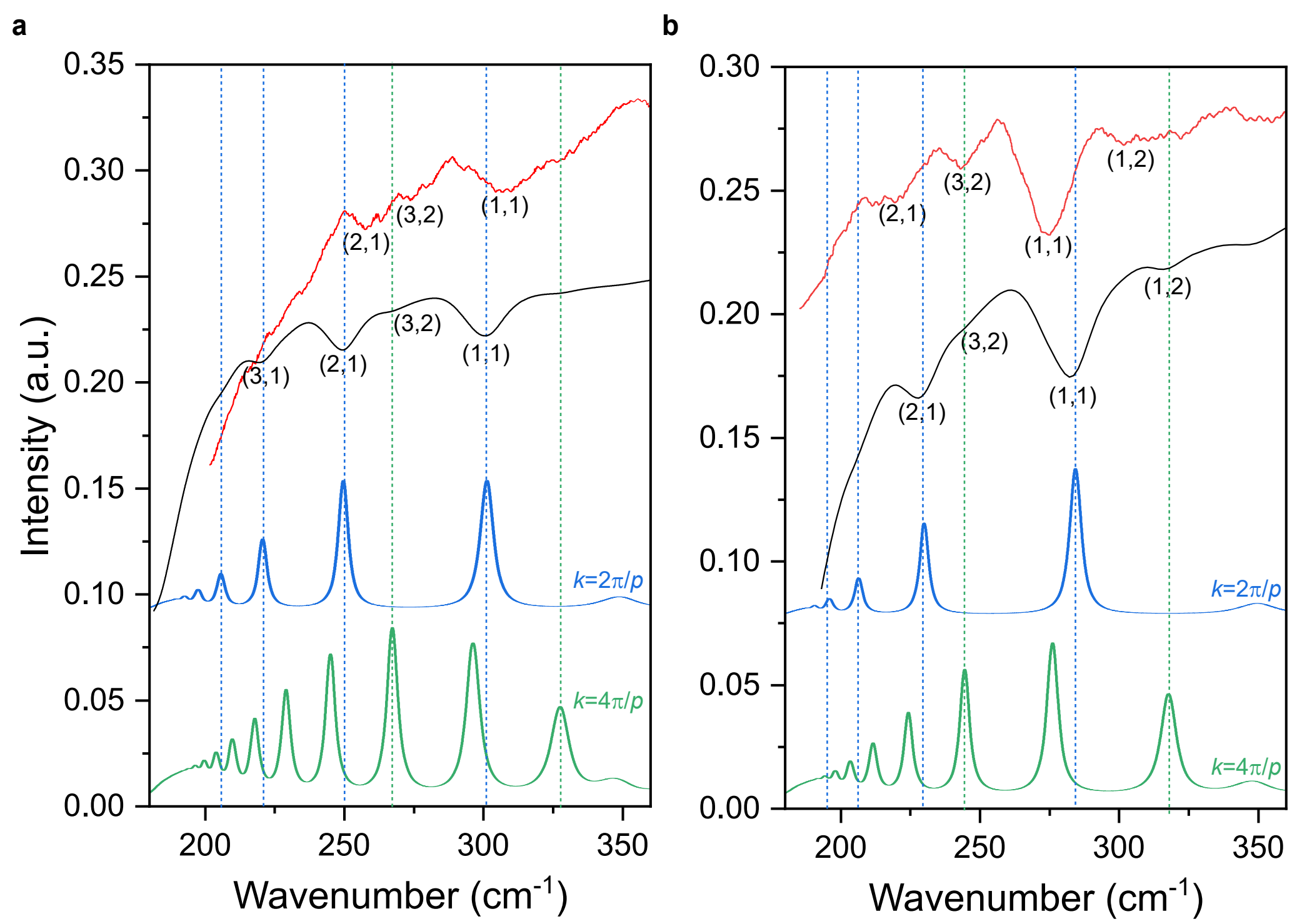

Figure S6. Resonance assignment based on calculated loss spectra. Comparison of the experimentally measured transmittance spectra with the calculated loss spectra evaluated at representative grating momenta for (a) $w = 250$ nm and (b) $w = 350$ nm. The blue and green curves correspond to the calculated loss spectra at $k = 2\pi/p$ and $k = 4\pi/p$, respectively, representing the first ($m = 1$) and second ($m = 2$) grating momentum orders. The vertical dashed lines mark the peak positions in the calculated loss spectra, and the corresponding experimental features are labeled as (n,m), where n denotes the polaritonic branch index and m denotes the grating momentum order. The agreement between the calculated loss peaks and the measured spectral features supports the assignment of higher-order polaritonic resonances.

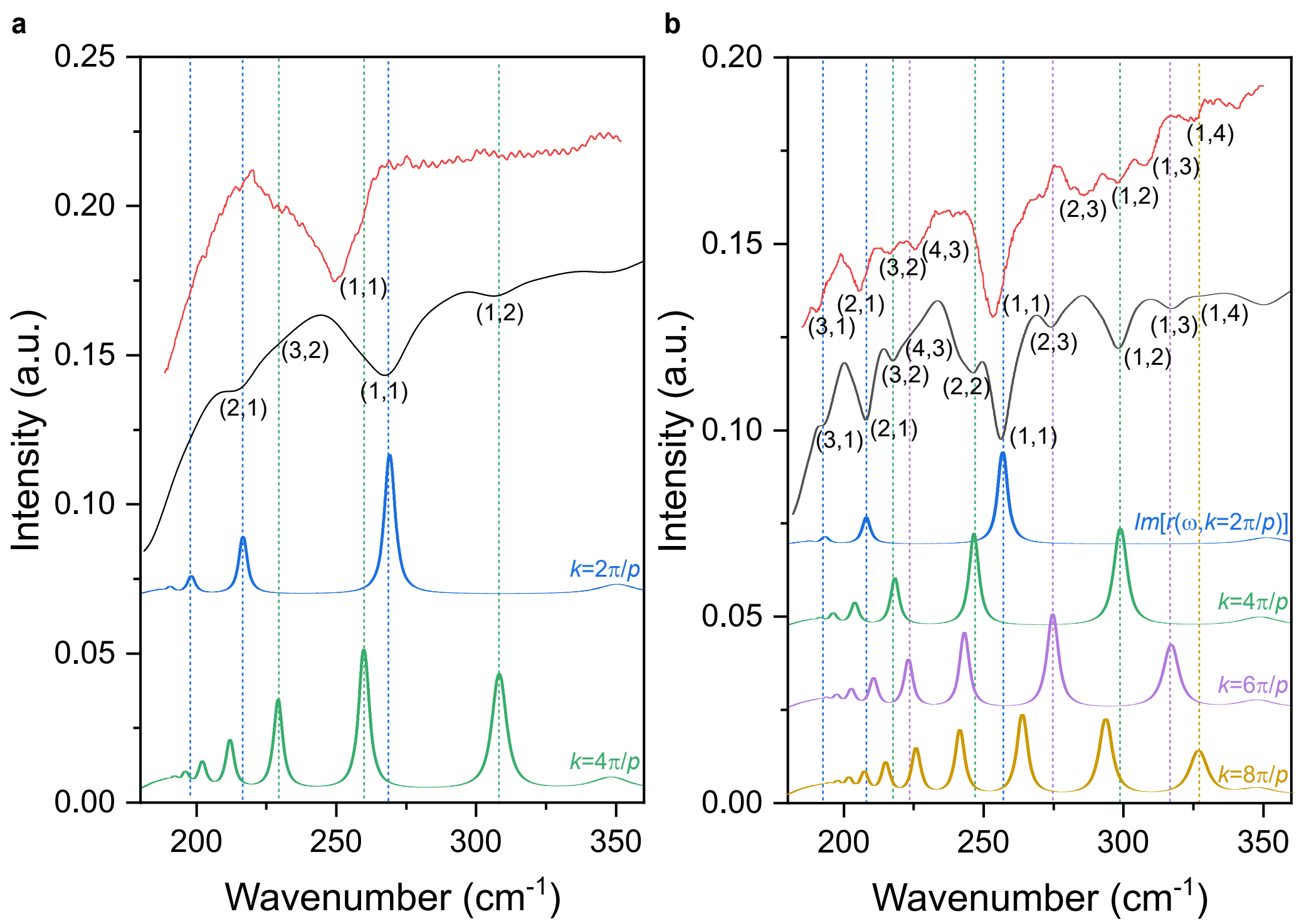

Figure S7. Resonance assignment based on calculated loss spectra. Comparison of the experimentally measured transmittance spectra with the calculated loss spectra evaluated at representative grating momenta for (a) $w = 450$ nm and (b) $w = 550$ nm. The blue, green, purple, and yellow curves correspond to the calculated loss spectra at $k = 2\pi/p$, $k = 4\pi/p$, $k = 6\pi/p$, and $k = 8\pi/p$ respectively, representing the first ($m = 1$), second ($m = 2$), third ($m = 3$), and fourth ($m = 4$) grating momentum orders.

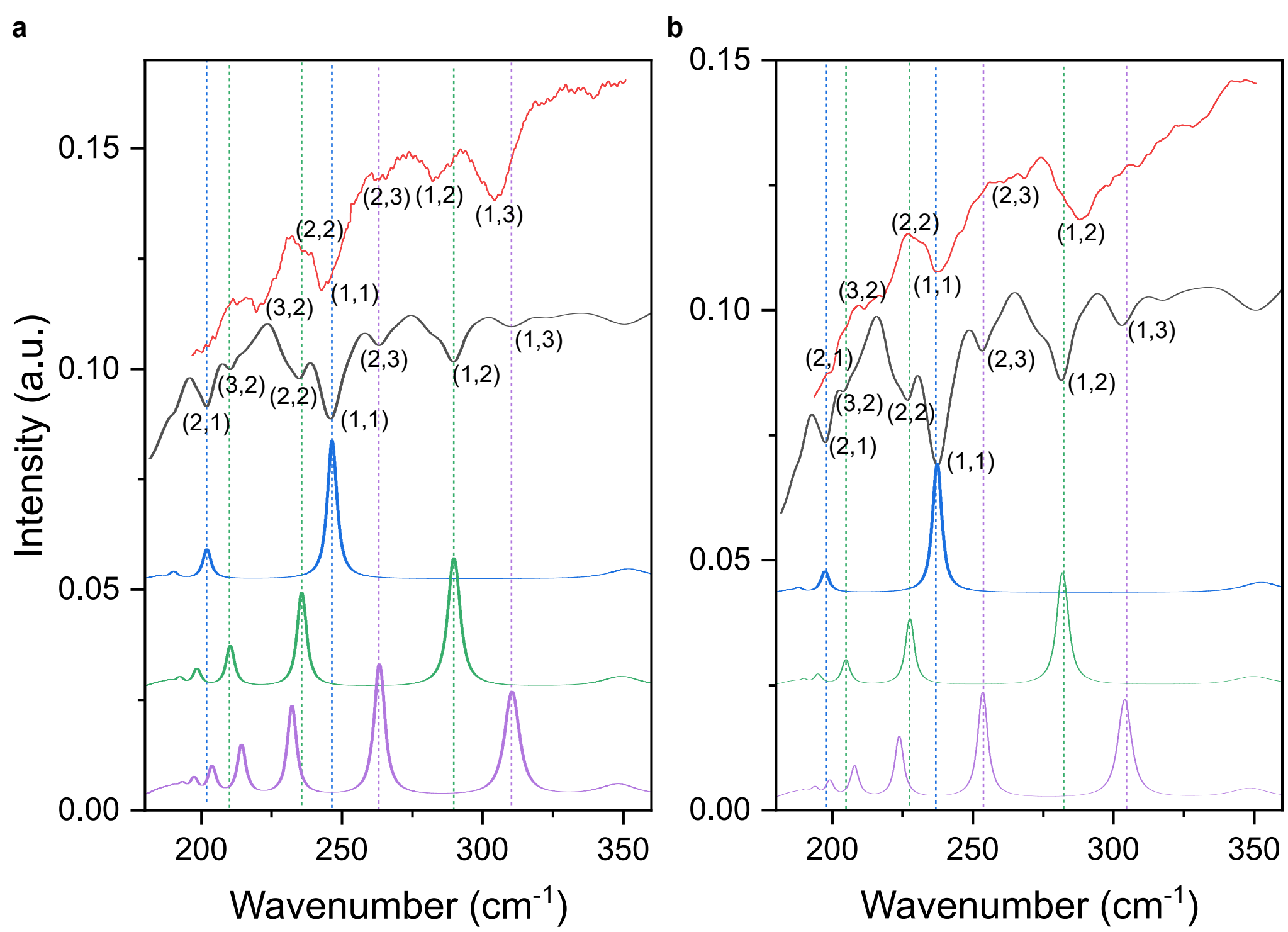

Figure S8. Resonance assignment based on calculated loss spectra. Comparison of the experimentally measured transmittance spectra with the calculated loss spectra evaluated at representative grating momenta for (a) $w = 650$ nm and (b) $w = 750$ nm.

Supplementary Note 2. Loss calculation.

The phonon–polariton dispersion is obtained in the form of a complex in-plane wavevector $q(\omega) = \Re(q) + i\Im(q)$ by numerically solving the complex-valued dispersion equation at each photon energy. From the calculated dispersion, the damping rate is extracted by combining the imaginary part of the wavevector with the group velocity.

The group velocity is then evaluated from the real part of the dispersion via a finite-difference approximation,

$$v_{\mathrm{g}}(\omega) = \frac{\mathrm{d}\omega}{\mathrm{d}\Re(q)} \approx \frac{\omega_{j+1} - \omega_{j-1}}{\Re(q_{j+1}) - \Re(q_{j-1})}, \tag{S14}$$

where the subscript j denotes discrete sampling points along the dispersion curve. In this calculation, the in-plane momentum q is expressed in units of nm^{-1} and is converted to m^{-1} when evaluating v_{g}.

The damping rate Γ is finally defined as

$$\Gamma(\omega) = 2\Im(q)\, v_{\mathrm{g}}, \tag{S15}$$

which has units of s^{-1} and quantifies the spatial attenuation of the phonon–polariton mode translated into a temporal decay rate through the group velocity. This definition follows directly from the exponential decay of the polariton amplitude, $\exp[-\Im(q)x]$, combined with the propagation velocity v_{g}.

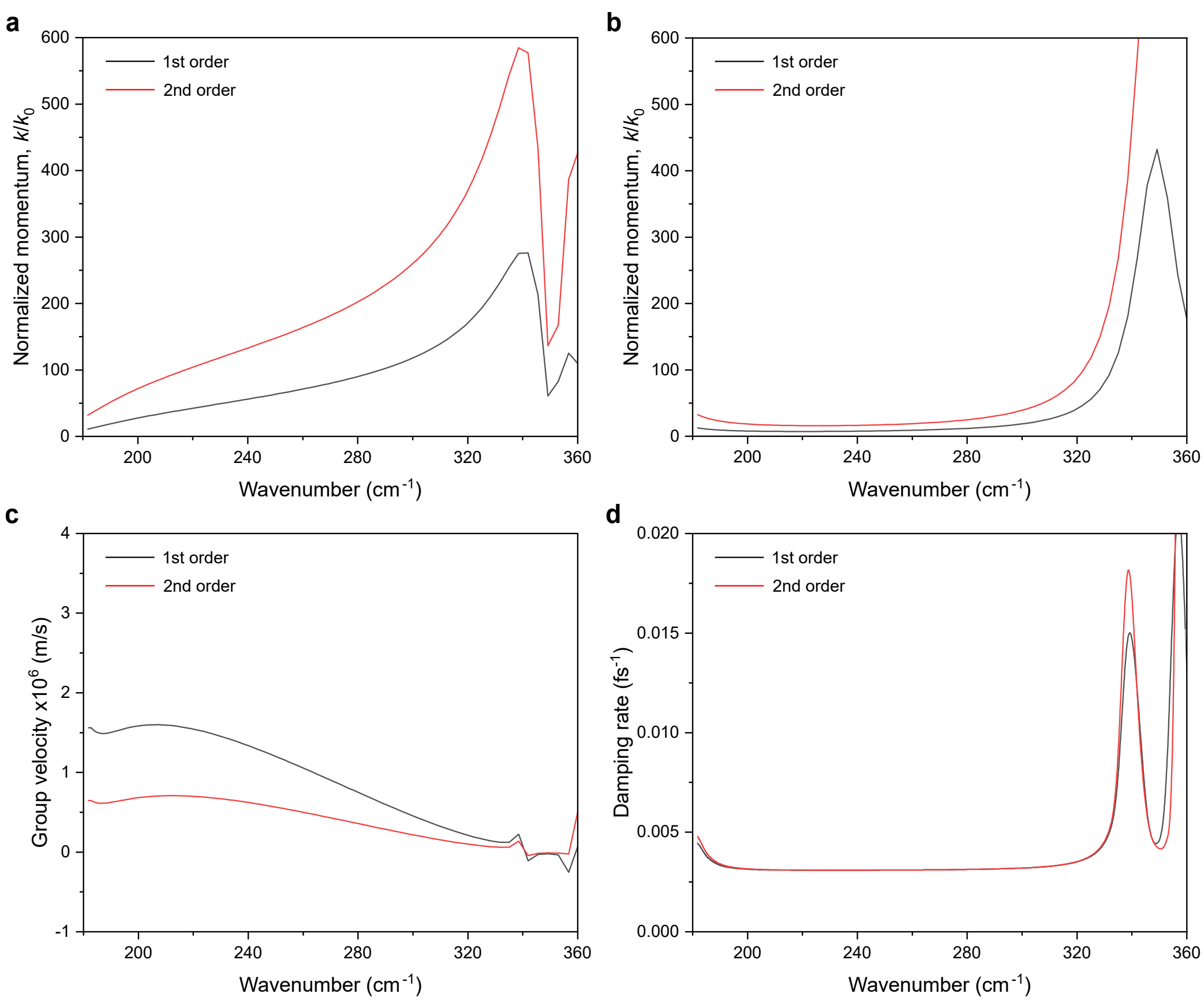

Figure S9. Loss analysis of ZrS_2 phonon polaritons. Numerically calculated (a) real and (b) imaginary parts of the normalized phonon–polariton momentum, as well as (c) the group velocity and (d) the damping rate of the phonon polaritons.

Table S1. Comparison of phonon-polariton lifetimes in representative polaritonic materials at room temperature.

Material	Spectral range	Measurement setup	Reported lifetime	Reference
ZrS_2	Far-IR ($181–351\ cm^{-1}$)	Far-field resonator	0.48 ps	This work
Isotope-enriched hBN	Mid-IR ($1394–1650\ cm^{-1}$)	Far-field resonator	0.57 ps	Ref.[4]
Isotope-enriched hBN	Mid-IR ($1359–1608\ cm^{-1}$)	Near-field imaging	2 ps	Ref.[5]
α-MoO_3	Mid-IR ($820–1010\ cm^{-1}$)	Near-field imaging	8 ps	Ref.[6]
Isotope-enriched α-MoO_3	Mid-IR ($958–1010\ cm^{-1}$)	Near-field imaging	13.9 ps	Ref.[7]
YVO_4	Mid-IR ($895–905\ cm^{-1}$)	Near-field imaging	5.55 ps	Ref.[8]
α-V_2O_5	Mid-IR ($765–1037\ cm^{-1}$)	Near-field imaging	5 ± 1ps	Ref.[9]

Supplementary References

1. Uchida, S.-i. & Tanaka, S. Optical phonon modes and localized effective charges of transition-metal dichalcogenides. *J. Phys. Soc. Jpn.* **45**, 153–161 (1978).
2. Glebko, N. *et al.* Electronic and vibrational properties of tis2, zrs2, and hfs2: Periodic trends studied by dispersion-corrected hybrid density functional methods. *The J. Phys. Chem. C* **122**, 26835–26844 (2018).
3. Laturia, A., Van de Put, M. L. & Vandenberghe, W. G. Dielectric properties of hexagonal boron nitride and transition metal dichalcogenides: from monolayer to bulk. *npj 2D Mater. Appl.* **2**, 6 (2018).
4. Lee, I.-H. *et al.* Image polaritons in boron nitride for extreme polariton confinement with low losses. *Nat. Commun.* **11**, 3649 (2020).
5. Giles, A. J. *et al.* Ultralow-loss polaritons in isotopically pure boron nitride. *Nat. Mater.* **17**, 134–139 (2018).
6. Ma, W. *et al.* In-plane anisotropic and ultra-low-loss polaritons in a natural van der waals crystal. *Nature* **562**, 557–562 (2018).
7. Zhao, Y. *et al.* Ultralow-loss phonon polaritons in the isotope-enriched α-moo3. *Nano Lett.* **22**, 10208–10215 (2022).
8. Liu, L. *et al.* Long-range hyperbolic polaritons on a non-hyperbolic crystal surface. *Nature* **644**, 76–82 (2025).
9. Taboada-Gutiérrez, J. *et al.* Broad spectral tuning of ultra-low-loss polaritons in a van der waals crystal by intercalation. *Nat. Mater.* **19**, 964–968 (2020).
